# Analysis of key genes of jasmonic acid mediated signal pathway for defense against insect damages by comparative transcriptome sequencing

**DOI:** 10.1038/srep16500

**Published:** 2015-11-12

**Authors:** Fengshan Yang, Yuliang Zhang, Qixing Huang, Guohua Yin, Kayla K. Pennerman, Jiujiang Yu, Zhixin Liu, Dafei Li, Anping Guo

**Affiliations:** 1 Key Laboratory of Molecular Biology of Heilongjiang Province, College of Life Sciences, Heilongjiang University, Harbin, Heilongjiang 150080, China; 2 Key Laboratory of Biology and Genetic Resources of Tropical Crops, Ministry of Agriculture, Institute of Tropical Bioscience and Biotechnology, Chinese Academy of Tropical Agricultural Sciences, Haikou, Hainan 571101, China; 3 Department of Plant Biology and Pathology, Rutgers, The State University of New Jersey, New Brunswick, New Jersey 08901, USA; 4 Department of Agriculture, ARS, Beltsville Agricultural Research Center, Beltsville, Maryland 20705, USA

## Abstract

Corn defense systems against insect herbivory involve activation of genes that lead to metabolic reconfigurations to produce toxic compounds, proteinase inhibitors, oxidative enzymes, and behavior-modifying volatiles. Similar responses occur when the plant is exposed to methyl jasmonate (MeJA). To compare the defense responses between stalk borer feeding and exogenous MeJA on a transcriptional level, we employed deep transcriptome sequencing methods following *Ostrinia furnacalis* leaf feeding and MeJA leaf treatment. 39,636 genes were found to be differentially expressed with *O. furnacalis* feeding, MeJA application, and *O. furnacalis* feeding and MeJA application. Following Gene Ontology enrichment analysis of the up- or down- regulated genes, many were implicated in metabolic processes, stimuli-responsive catalytic activity, and transfer activity. Fifteen genes that indicated significant changes in the *O. furnacalis* feeding group: *LOX1, ASN1, eIF3, DXS, AOS, TIM, LOX5, BBTI2, BBTI11, BBTI12, BBTI13, Cl-1B, TPS10, DOX*, and *A20/AN1* were found to almost all be involved in jasmonate defense signaling pathways. All of the data demonstrate that the jasmonate defense signal pathway is a major defense signaling pathways of Asian corn borer’s defense against insect herbivory. The transcriptome data are publically available at NCBI SRA: SRS965087.

Stalk borers are global insect pests of both agricultural crops and weedy species. They include many species of moth larvae that feed on the stalks of poaceous plants, causing yield loss, early leaf senescence, disruption of metabolite transport and increased susceptibility to pathogen infection^1^. General and specific host responses to insect herbivory may depend on the mode of attack (i.e. piercing versus chewing), oral secretion components, volatile signaling from neighboring plants, and abiotic and biotic stresses. One of most destructive and worldwide widespread species of corn stalk borers in Asia is *Ostrinia furnacalis* (Guenée), the Asain corn borer, which enters the stalk and burrows in the pith tissues. An infestation can lead to 10%~30% crop yield loss^2^. In corn (*Zea mays*) lines, thicker cell walls, higher xylose and diferulate (plant cell wall cross-linkers) concentrations, and internode length correlate with constitutive resistance to stalk borers^3^,^4^, though an earlier study indicates that stronger structural traits may not necessarily confer resistance^5^. Induced defensive mechanisms tend to involve direct interference with insect growth and development via toxic proteins and metabolites, strengthening of structural tissues, and attraction of predators of the insect^6^.

Such induced responses in corn have been identified by several microarray, qRT-PCR, and metabolite assays. Primarily, these studies find that certain types of genes and regulatory elements are up-regulated after insect herbivory: transcription factors^7^,^8^, defensive protein production (including anti-disgestives and chitinases)^1^,^7^, ethylene (ET), and jasmonic acid (JA) perception, regulation and biosynthesis^7^,^9^, and terpenoid phytoalexins synthesis^10^. For instance, genes controlling the synthesis of insecticidal benzoxazinoids and terpenoid phytoalexins are up-regulated within 24 h of stalk borer feeding, as are the concentrations of these compounds^11^,^12^. Responses may also include strengthening of the cell walls. Genes involved in cell wall organization, such as *β-1,3-glucanase* and *cinnamoyl CoA reductase 2*, are up-regulated in corn in response to the stalk borer *Sesamia nonagrioides*^1^. The products of these genes are suggested to be involved in cellulose degradation and increased lignin content, respectively. Corn cell walls after borer attack are known to contain more lignin^1^.

JA production appears to be responsible for a large portion of the differential regulation of defensive genes and regulatory elements. It is well-known that exposure to the signaling hormone can “prime” plants against biotic and abiotic stresses, improving tolerance^13^. Plants respond to exogenous methyl jasmonate (MeJA) with a myriad of inducible defense responses including the production of toxic metabolites and anti-digestive proteins which harm feeding insects. These similarities to defense responses against insect herbivory make it possible to stimulate plant resistance before insect attack. Such a strategy may become useful in future pest management strategies^14^, employing MeJA or other stimuli of JA pathways such as hexanoic acid^15^.

Necessarily, a plant’s transcriptome and proteome has to be significantly modified to induce and support the production of defensive metabolites and proteins. However, to our knowledge, transcriptome comparisons of corn plants responding to stalk borer attack or MeJA have not been reported. We previously completed a proteomic analysis of corn subjected to MeJA treatment^16^. Many of the differentially-accumulated proteins had energy-related, regulatory, and defensive functions. The aims of the current work was to investigate the similarities and differences between Asian corn borer feeding and methyl jasmonate induction, to explore the synergistic or anatagonistic effects between them, and to find which defense mechanisms are more important during plant defense response. From this, we hoped to provide more scientific information for future work such as the study of the functions of important genes or proteins in the plant defense pathway. Here, we report a comprehensive transcriptome profiling analysis of inducible defense genes involved in response to MeJA, Asian corn borer attack, and MeJA and Asian corn borer attack, to identify transcript modification which may affect protein accumulation.

## Results

### RNA sequencing results

One control (Corn1) and three treated (CornJA1, MeJA application only; CornOf2, *O. furnacalis* feeding only; CornJAOf2, MeJA application and *O. furnacalis* feeding) sample groups were subjected to RNA sequencing using the Illumina HiSeq 2500 sequencing platform. High-quality base reads were obtained in this study: all the raw reads and base counts, and their qualities are listed in Table 1. We trimmed all the raw reads by removing low-quality reads and adapters. The Q20 scores of clean bases reached over 97% in these three treatments. After raw read trimming, the mapped ratio reached at least 81% (Table 1).

### Transcriptome analysis of corn leaves challenged with Asian corn borer feeding and/or MeJA

**Figure 1:**
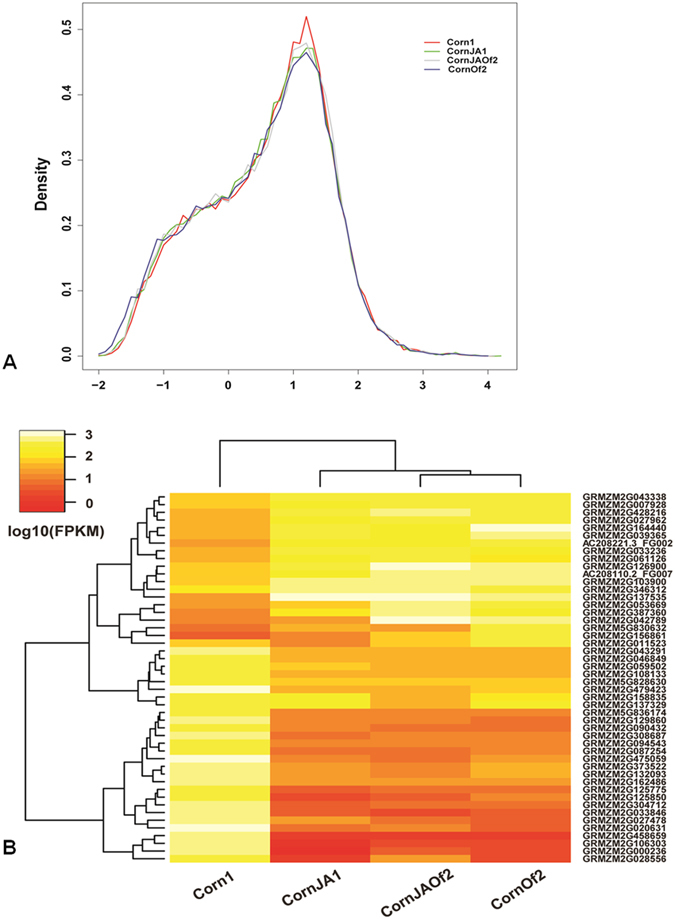
FPKM distribution and clustering of differentially expressed genes in each treatment. (**A**) Distribution of genes according to FPKM values in CornJA1, CornOf2, and CornJAOf2 treatments and control group. Log_10_ (FPKM) indicates the log_10_ based FPKM value; genes with FPKM values between 10 and 100 are predominant. (**B**) Clustering of differentially expressed genes in each treatment. Forty-eight genes with FPKM ≥300 are shown. The color scale indicates gene expression level. Two major subgroups of genes were defined according to gene expression patterns in different treatments.

The FPKM distributions of Corn1, CornJA1, CornOf2, and CornJAOf2 are shown in Fig. 1A. In all four sample groups, the majority of detected genes have FPKM values ≥10. The relationships among differentially expressed genes (DEGs) were revealed by hierarchical cluster analysis. Figure 1B depicts the cluster analysis of 48 genes with FPKM ≥300 and a heat map showing their expression levels.

**Figure 2:**
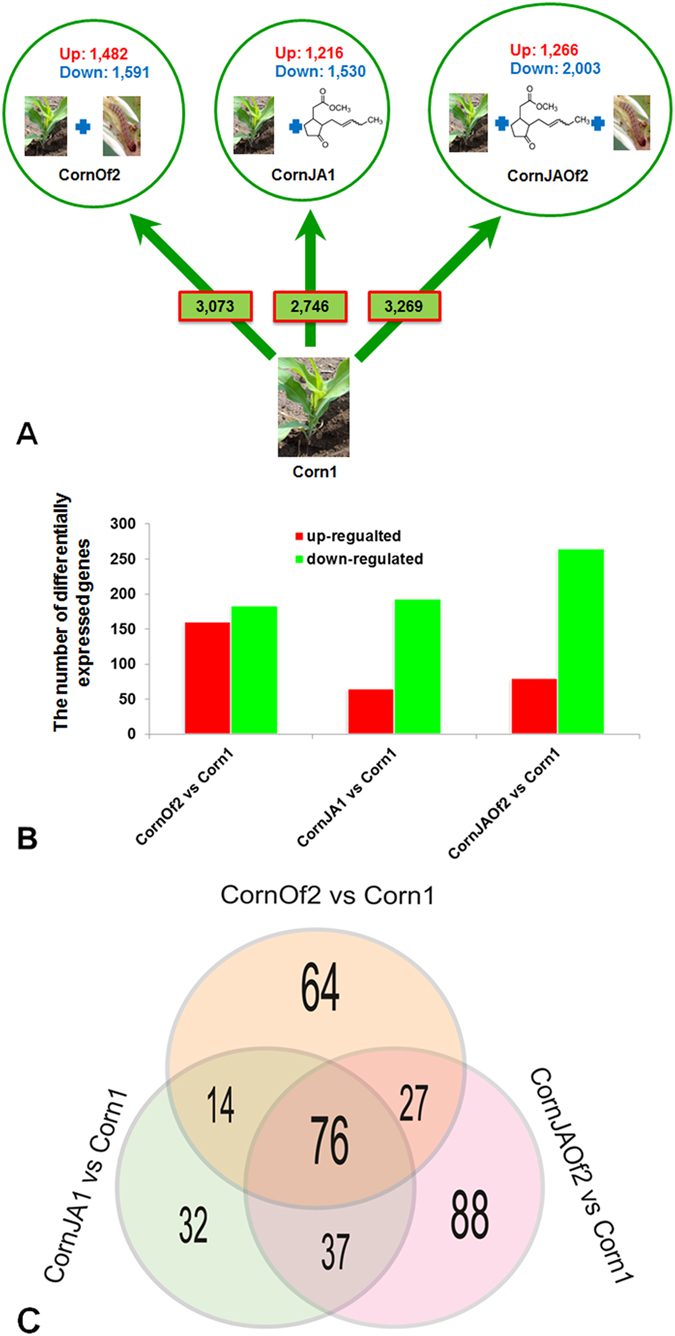
Number of differentially expressed genes in the three treatments. (**A**) Differentially expressed genes in corn under three different treatments with FDR ≤ 0.05 and change fold ≥2, and (**B**) differentially expressed genes in corn under three different treatments with FDR ≤ 0.00l and change fold ≥2, and the red and green colors denote the up-regulated and down-regulated genes, respectively, and (**C**) the Venn diagram of common genes induced after three treatments, the significantly different (FDR ≤ 0.05) genes were defined as transcripts with fold changes ≥2 based on gene expression levels between CornJA1 and Corn1, between CornOf2 and Corn1, between CornJAOf2 and Corn1, respectively.

With false discovery rate (FDR) ≤0.05 and log_2_ ratio ≥1 to screen DEGs, a total of 39,636 DEGs were detected including 23,323 up-regulated genes and 16,313 down-regulated genes in all comparisons to the control group. Among these genes, 27,170 genes could be annotated to *Arabidopsis* (68.55%). 3,073 (1,482 up-regulated and 1,591 down-regulated), 2,746 (1,216 up-regulated and 1,530 down-regulated), and 3,269 (1,266 up-regulated and 2,003 down-regulated) DEGs were significantly changed at least two-fold in CornOf2, CornJA1, and CornJAOf2 treatments, respectively, compared to control group (Fig. 2A). After setting more strict conditions (FDR ≤ 0.001 and log_2_ ratio ≥2) to screen DEGs, 343 (160 up-regulated and 183 down-regulated), 258 (65 up-regulated and 193 down-regulated), and 345 (80 up-regulated and 265 down-regulated) DEGs were significantly changed at least four-fold in CornOf2, CornJA1, and CornJAOf2 treatments, respectively, compared to the control group (Fig. 2B).

Across all the three treated samples, most DEGs were assigned to GO terms such as biological process, metabolic process, response to stimulus, catalytic activity, transferase activity, and oxidoreductase activity. More DEGs in CornJA1 group were also assigned the terms organonitrogen compound metabolic process, response to abiotic stimulus, extracellular region, compared to the CornOf2 group. On the other hand, a slightly more diverse set of GO terms are used to describe DEGs found in the CornOf2 and CornJAOf2 groups. GO assignment highlights some of the differences in transcriptome response to the different treatments.

The two most enriched KEGG pathways were metabolic pathways and biosynthesis of secondary metabolites. More DEGs from the CornJAOf2 group were mapped to those pathways compared to the other two treatment groups, indicating a positive interaction between MeJA application and Asian corn borer feeding for these pathways. Conversely, glutathione metabolism appeared to be negatively affected by the combination of MeJA and insect feeding.

Counts of overlapping DEGs among the three different treatments are shown in Fig. 2C. Altogether, 76 DEGs were detected in all three comparisons. Using KEGG, 15 significantly changed DEGs were annotated (Table S7). These genes are likely to be involved in JA and salicylic acid (SA) signaling pathways. The top thirteen genes are all associated with the defense genes in JA signal transduction and nine of them were chosen to validate their expression levels by qRT-PCR. Two genes (GRMZM2G053669 and GRMZM2G301904) are related to SA signal transduction. GRMZM2G179092 and GRMZM2G156861 were found to be the most significantly up-regulated in CornOf2 and CornJAOf2 treatments. These two genes are terpene synthase 10 (TPS10) and lipoxygenase 1 (LOX1), respectively, and are members of the prenyltransferase protein superfamily. TPS10 is a critical enzyme in terpene biosynthesis^17^,^18^ and had the most transcript accumulation after Asian corn borer feeding, and similar changes after the other treatments. This indicated that terpene production significantly changed for active participation against insect herbivory. Other genes with large changes are known or suspected to play roles in resistance to pests^19^,^20^,^21^,^22^,^23^,^24^,^25^,^26^,^27^.

### Validation of differential gene expression using qRT-PCR

**Figure 3:**
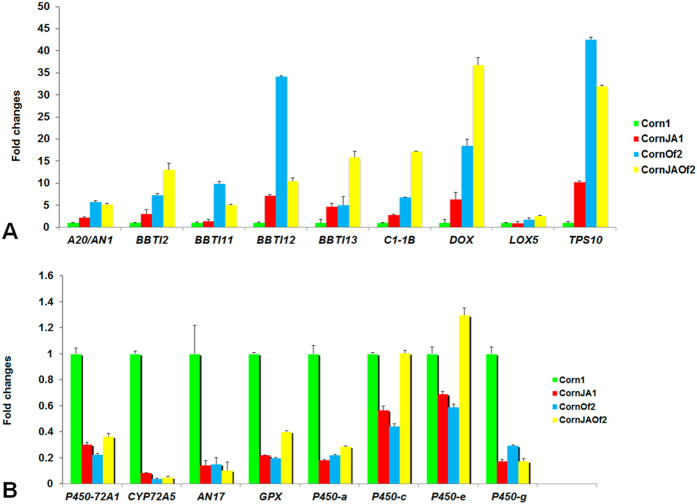
The qRT-PCR evaluations of the genes involved in JA defense response. The (**A**) up- and (**B**) down-regulated genes in JA defense response are shown. Note: A20/AN1, zinc finger A20 and AN1 domain-containing stress-associated protein; BBTI2/11/12/13: Bowman-Birk type inhibitor 2/11/12/13; CI-1B: subtilisin-chymotrypsin inhibitor CI-1B; DOX: α-dioxygenase; LOX5: lipoxygenase 5; TPS10: terpene synthase 10; GAPDH: internal reference gene; CYP72A5, P450-72A1, P450-a, P450-c, P450-e, and P450-g: different transcripts of cytochrome 450; AN17: AN1-like zinc finger domain containing protein AN17; GPX: glutathione peroxidase domain containing protein. The up-regulation of P450-e in CornJAOf2 treatment may be caused by error.

To confirm the DEGs induced by MeJA treatment and/or Asian corn borer feeding, we performed qRT-PCR analysis of nine genes involved in JA defense response to evaluate our transcriptomic analyses: lipoxygenase 5 (*LOX5*), Bowman-Birk type bran trypsin inhibitor precursors *BBTI2, BBTI11, BBTI12, BBTI13*, subtilisin-chymotrypsin inhibitor CI-1B (*Cl-1B*), terpene synthase 10 (*TPS10*), α-dioxygenase (*DOX*), and zinc finger A20 and AN1 domain-containing stress-associated protein (*A20/AN1*). The descriptions and comparisons of RNA-Seq and qRT-PCR results of these nine genes are summarized in Table S8. We also randomly chose eight significantly down-regulated transcripts to check if their transcript accumulation really decreased. All of the primers are shown in Table 2. All assayed genes exhibited the expected positive or negative fold changes in qRT-PCR reactions (except for LOX5 in CornJA1) (Fig. 3). Average fold change differences in these genes were calculated and were found to mostly correspond with the transcriptomic analyses.

### Analysis of transcript alternative splicing and SNPs

We also analyzed all possible alternative splicing events and SNPs. Compared to the control group, CornOf2 treatment showed the largest changes in alternative splices and SNPs: 3′ alternative splices (38.14%), alternative 3′ UTR splices (24.42%), 5′ alternative splices (32.84%), 5′ UTR splices (26.57%), retained introns (47.37%), and other possible alternative splices (49.61%); followed by CornJA1 treatment and CornJAOf2 (Tables 3 and 4). For skipped exons, CornJAOf2 showed the largest change (−39.53%) compared with CornJA1 (−35.59%) and CornOf2 (−13.79%) (Table 3). All the data showed that Asian corn border feeding greatly affected corn’s alternative splicing. SNPs were identified by SAMtools and VarScan software. Compared with the control group, SNPs in CornOf2 (frequency per kb) showed the highest changes in C/T and A/G transitions (21.95% each) and A/T (21.05%), A/C (16.67%), T/G (16.67%) and C/G (14.29%) in transversions, followed by the CornJAOf2 and CornJA treatment groups (Table 4).

## Conclusion and Discussion

JA signaling, SA signaling, and ET signaling are usually implicated in plant defenses. These signaling pathways communicate with one another and coordinate the modification of the transcriptome to resist pests with minimal disruption of growth and development^28^. For example, GRMZM2G301904, a eukaryotic translation initiation factor 3C that plays an important role in protein translation processes and is mainly involved in defense response to plant viruses in an SA-induced defense signaling pathway^29^, was found to be up-regulated in this study. However, our data indicated that the JA signaling pathway was the main defense response in maize. Fewer DEGs involved in SA and other defense signaling pathways were detected. Asian corn borer feeding mainly induces JA defense pathways for an increased chance of plant survival. This does not exclude the possible roles of complex interactions among phytohormones.

Exogenous MeJA is known to induce defense mechanisms in plants similar to insect attacks. By Illumina sequencing technology, we successfully obtained four large groups of transcriptome data that almost completely cover all the DEGs responsive to MeJA, Asian corn borer feeding, and both. Compared to the control group, nearly 40,000 DEGs were identified. Of those, around 3,000 DEGs were significantly changed at least two-fold for each treatment group. Among all the DEGs in the three treatments, the relative fold change of each gene in CornOf2 treatment group was usually the highest followed by the CornJAOf2 and CornJA1 groups. This might be because Asian corn borer feeding caused greater damages to the corn host, including physical injuries and loss of nutrients, which further prompted the host to produce large amounts of defensive substances which strongly resist pest infestation. Additionally, the corn borer may have released counteractive inducers which lower or raise plant resistance to insect herbivory. Corn earworm (*Helicoverpa zeae*) secretes glucose oxidase which interferes with nicotine accumulation in tobacco leaves^30^. The saliva of borers *Pieris brassicae* and *Spodoptera littoralis* are known to down-regulate wound-inducible genes of proteinase inhibitors and transcription factors in *Arabidopsis*^31^. Dafoe *et al.* found that the European corn borer (*Ostrinia nubilalis*) excretes auxin indole-3-acetic acid in its waste which may reverse JA-induced mechanisms in the feeding tunnel^11^. Therefore, insect feeding could result in a larger amount of gene expression changes compared to the other treatments, but certain genes may be relatively less expressed. Occasionally, we found that the fold change of some genes in the CornJAOf2 group was greater than that of the CornOf2 group. This may be caused by synergistic effects of Asian corn borer feeding and MeJA induced defense responses^30^,^31^,^32^.

Our results show that corn responds to Asian corn borer feeding through a considerable amount of transcriptional restructuring and activation of defense-related pathways. Most of the up-regulated transcripts have been previously reported to be responsive to insect feeding. This includes AOS which catalyzes the dehydration of hydroperoxide to an unstable allene oxide in the JA biosynthetic pathway^33^. Two WRKY transcription factors were also up-regulated which are suggested to be involved in the JA-signaling pathway in *Arabidopsis*^34^. In tomato, WRKY1 expression is induced by MeJA, as well as biotic and abiotic stresses, and increases tomato resistance to *Phytophthora*^35^,^36^. MeJA application also showed a similar effect: a large number of genes encoding plant protease inhibitors (PIs) were found to be increased in response to MeJA treatment. After insect herbivory, PIs can have defensive functions in plants by interfering with normal feeding and digestion of insects. We have identified four BBI DEGs (BBIT2/11/12/13) in this study. BBIs have also been found to be differently expressed in corn after insect feeding in other studies^1^,^8^. Phytoalexins have also been previously reported as being involved in resistance responses in maize^10^. In this study, nine genes earlier shown to be involved in JA synthesis and production of terpenoid and phytoalexins were demonstrated to be significantly up-regulated by corn borer feeding and/or MeJA application. Previous studies from other groups and our own indicate that PIs, terpenoids, and phytoalexins are likely to be central to the maize defense response and that these metabolites may offer alternatives for plant breeding programs and transgenic approaches towards developing insect-resistant crops^37^,^38^.

**Figure 4:**
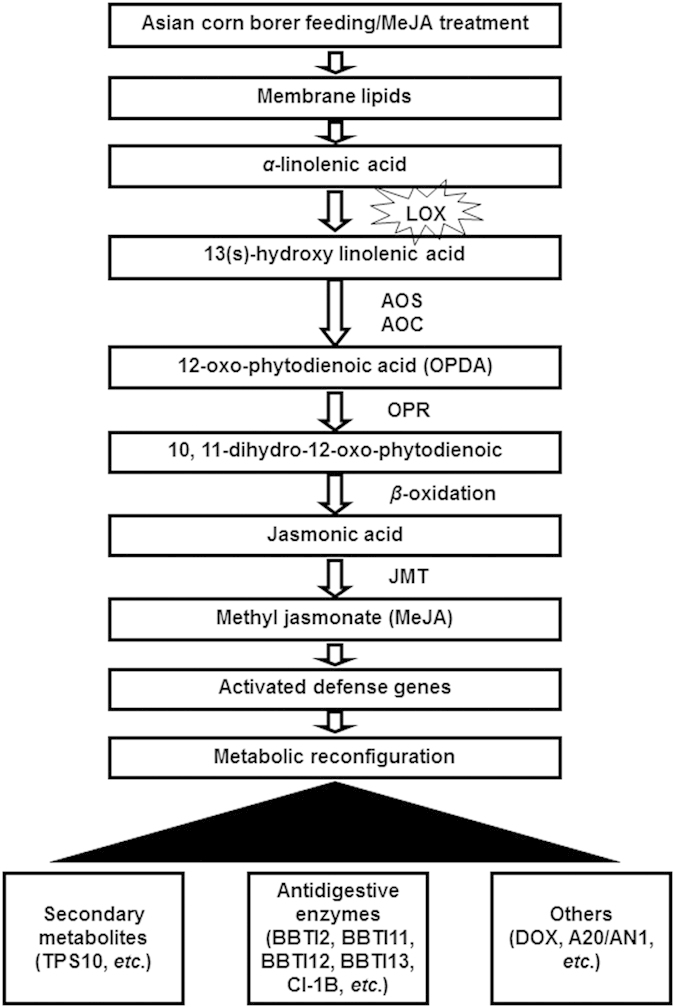
A hypothetical model of corn borers/MeJA reponse in maize. Note: AOC: allene oxide cyclase; OPR: 12-oxo-phytodienoic acid reductase; JMT: jasmonic acid carboxyl methyltransferase.

Transcriptome sequencing is a very efficient way to detect the alternative splicing and SNPs. In this study, we detected a large increase of alternative splicing and SNPs of the transcripts of the CornOf2 treatment group. Studies have shown that alternative splicing can greatly affect the evolution, development, adaptation or resistance of plants and insects with or without stressors^39^. Fabrick *et al.* reported that alternative splicing and highly variable cadherin transcripts are associated with field-evolved resistance of pink bollworm (*Pectinophore gossypiella*) to Bt cotton. Altogether, the group detected 19 transcript isoforms in eight alleles^40^. Using diagnositic PCR, Cui *et al.* reported two alternative splicing sites in the *O*fRyR gene of *O. furnacalis*; two exons are present during all stages of development while a third is not detected in the egg, suggesting different purposes^39^. Similarily, the large number of alternative splicing in our study indicates that products of the same defensive genes may have distinguishable functions and efficiencies. Perhaps, alternative splicing and SNPs and their effects on RNA stability and degradation rate can provide answers to the apparent misalignment of accumulated transcripts and proteins. Based on the current and previous studies, a hypothetical model of corn borer/MeJA reponse in maize was constructed (Fig. 4).

To our knowledge, this study provides the first insights into global gene expression changes of a plant in response to challenge by Asian corn borer feeding and/or MeJA inducement. By analyzing the changes in gene expression in the host plant, our analysis provides important new information on the basis of resistance to the agricultural pest on a molecular level. This also complements our previous proteomic work with corn exposure to exogenous MeJA^16^. As expected, we found that many key defense genes were up-regulated, demonstrating that general plant defense mechanisms were activated, including the JA biosynthetic pathway, proteinase inhibitors, and defense-related transcription factors. However, with this work, further questions about how well RNA levels translate to protein levels have been raised.

## Materials and Methods

### Insect resource

A colony of *O. furnacalis* was maintained at College of Life Sciences, Heilongjiang University (Heilongjiang province, China) on an artificial diet (5 g vitamin C, 40 g yeast extract, 50 g barley powder, 60 g soybean powder, 14 mL ethylic acid (36%), 20 g agar powder, 1 g benzoic acid, 3 g sodium benzoate in 1,000 mL ddH_2_O). The Asian corn borers were reared at 28 °C under a 14 h light: 10 h dark photoperiod.

### Plant materials, Asian corn borer feeding and MeJA treatment

Corn seeds of variety Longdan46 were provided by the Maize Research Institute of Heilongjiang Academy of Agricultural Science and cultivated in a greenhouse at 25 ± 1 °C, 60% humidity, and a 14 h light: 10 h dark photoperiod. Corn plants during the three-leaf stage of development were challenged with Asian corn borer larvae with or without MeJA treatment. For each treatment, 5 plants with 4~5 leaves were used and the entire experiment was repeated 3 times. Whole plants were covered with transparent plastic bags (38 cm in length and 25 cm in width). For the control group, corn plants were treated with 5 mL of 0.5% ethanol/water (v:v) solution (labelled as “Corn1”). For the MeJA treatment group, corn plants were treated with 5 mL of a 225 μM MeJA solution (95% MeJA diluted in dehydrated alcohol to reach final alcohol at 0.5%; labelled as “CornJA1”). For the Asian corn borer feeding treatment group, 20 1^st^ instar larvae of *O. furnacalis* were evenly distributed on the leaves of each corn plant (labelled as “CornOf2”). For the MeJA and Asian corn borer feeding treatment group, corn plants were treated with 5 mL of a 225 μM MeJA solution and were also fed on by 20 1^st^ instar larvae of *O. furnacalis* (labelled as “CornJAOf2”). Application of both MeJA and *O. furnaclis* gave us the opportunity to explore the complex interactions between these two treatments and magnification of any effects. The third leaf counted from the bottom of each plant was selected and immediately frozen in liquid nitrogen and stored at −80 °C for later RNA extraction.

### The cDNA library construction and sequencing

Total RNA was isolated and purified from leaf tissue using the TRIzol Plus RNA Purification Kit according to the manufacturer’s instructions (Life Technologies, http://www.lifetechnologies.com). To remove any traces of genomic DNA from RNA extractions, the RNA was treated with RNase-Free DNase (Promega, http://www.promega.com) as instructed by the manufacturer. Total RNA integrity and quantity were evaluated using the 2100 Bioanalyzer RNA 6000 Kit (Agilent Technologies, http://www.agilent.com/home) and the Invitrogen Qubit RNA Kit (Life Technologies, USA). RNA samples had integrity values (RIN) between 7.6 and 9.0. A minimum of 15 μg of purified total RNA per sample was prepared for transcriptome analysis. Libraries were prepared for sequencing according to TruSeq protocols (Illumina, http://www.illumina.com). The TruSeq RNA Sample Prep Kit was used to isolate mRNA from about 5 μg of total RNA using oligo-d(T)25 magnetic beads, shear it with ions to 2 × 100 bp fragments and synthesize cDNA. We then used the TruSeq PE Cluster Kit v3 to perform end repair, add an ‘A’-base to the blunt ends, and ligate the cDNA to paired-end adaptors. The cDNA samples were amplified through 15 cycles of PCR. Target bands were recycled using 2% Certified Low Range Ultra Agarose gel (Bio-Rad, http://www.bio-rad.com). Amplification products were quantified using PicoGreen (Life Technologies) and a TBS-380 Mini-Fluorometer (Promega) and loaded on an Illumina cBot system for cluster generation by bridge PCR amplification. Sequencing was performed on an Illumina HiSeq 2500 sequencing platform.

### Transcriptome assembly and bioinformatics analysis

For each read, we followed four steps to obtain the clean reads: (1) remove adapters and those reads that were not successfully inserted because of adapter self-ligation; (2) trim off the low quality 3′ ends (Q ≤ 20), if the quality value of the remaining sequence was still less than 10, then the entire sequence was deleted; (3) remove reads with N ratios higher than 10%; (4) delete sequences with lengths below 20 bp. SeqPrep (https://github.com/jstjohn/SeqPrep) and Sickle (https://github.com/najoshi/sickle) at default parameters were used to trim all the raw reads. After quality control, we mapped all the clean reads to the reference genome (http://ftp.jgi-psf.org/pub/compgen/phytozome/v9.0/Zmays) using TopHat (http://tophat.cbcb.umd.edu/)^41^. Duplicate reads and gene coverage degree were analyzed by RSeQC-2.3.2 (http://rseqc.sourceforge.net)^42^. We calculated gene expression values by the read/fragments per kilobase of exon per million fragments mapped (RPKM/FPKM) formula using Cuffdiff (http://cufflinks.cbcb.umd.edu/)^43^,^44^,^45^. In order to control the Type I error rate and get true differentially expressed genes (DEGs), the *p*-value was rectified using the False Discovery Rate (FDR) control method. Here, FDR was the expectation of false discovery rate: FDR = E (V/R), and R is the number of selected DEGs, V is the number of false positive DEGs, and E is expectation. Both the FPKM ratio and FDR value in all treatments were calculated. Genes with an FPKM ratio ≥2 and FDR ≤ 0.05 were considered to have significantly different expression levels between treatments. We analyzed the gene relationships of the different treatments and identified the overlapping DEGs using VennDiagram^46^.

Gene Ontology (GO, http://www.geneontology.org), is the comprehensive database established by the Gene Ontology Consortium. We use Blast2GO (http://www.blast2go.com) to annotate all the clean reads with GO terms^47^. Based on KEGG database (Kyoto Encyclopedia of Genes and Genomes, http://www.genome.jp/kegg/), NCBI blastx/blastp 2.2.24+ was used to align all the genes with database genes^48^. Cluster analysis of significantly expressed genes/transcripts was performed using the distance calculation methods Spearman (different treatments) and Pearson (within one treatment). The clustering method was hcluster (http://pypi.python.org/pypi/hcluster). GO functional enrichment analysis was performed using GOatools (http://github.com/tanghaibao/GOatools) by Fisher’s exact test. Four multiple testing methods (Bonferroni, Holm, Sidak, and false discovery rate) were used to avoid the false positive rate^49^ and to correct *p*-values^50^,^51^,^52^,^53^; significant enrichment in GO analysis was considered when the *p*-value was smaller than 0.05. The pathway enrichment analysis of DEGs was performed using KOBAS (http://kobas.cbi.pku.edu.cn/home.do)^54^. Cluster of Orthologous Groups of proteins (COG) is the database for gene/protein orthologous classification (http://www.ncbi.nlm.nih.gov/COG/). The identified genes were compared with the COG database to predict gene or proteins’ function.

Multivariate Analysis of Transcript Splicing (MATS) (http://rnaseq-mats.sourceforge.net) was used to detect differential alternative splicing events from RNA-Seq data^55^. New transcripts were identified using Cufflinks (http://cole-trapnell-lab.github.io/cufflinks/)^56^. Single nucleotide polymorphisms (SNPs) were identified using SAMtools (http://samtools.sourceforge.net)^57^ and VarScan v.2.2.7 (http://varscan.sourceforge.net).

### Data statistical analysis and accessibility

All the data were analyzed using JMP software version 5.0 (SAS Institute Inc., http://www.sas.com). F-value (*p* = 0.05) was considered as significantly changed. The sequencing reads were submitted to the NCBI SRA and can be accessible via NCBI BioProject accession SRS965087.

### Validation of transcriptome analysis using qRT-PCR

Total RNA (1 μg) was reverse-transcribed using the PrimeScript RT Reagent Kit with gDNA Eraser (Takara Bio Inc., http://www.takara-bio.com). Genomic DNA was removed in a 10 μL reaction system: 2.0 μL of 5X gDNA eraser buffer, 1.0 μL of gDNA Eraser, ~1 μg of total RNA, and added RNase-free water to 10 μL. The cDNA was synthesized in a 20-μL reaction system as follows: 4.0 μL of 25 mM MgCl_2_, 2.0 μL of reverse transcription 10X buffer, 2.0 μL of 10 mM dNTP mixture, 1.0 μL of oligo(dT)15 primer, 2.0 μL of extracted total RNA (~1 μg), and added nuclease-free water to a final volume of 18.5 μL. The mixture was pre-heated at 70 °C for 10 min and chilled on ice for 1 min followed by addition of 0.5 μL recombinant RNasin ribonuclease inhibitor and 1.0 μL of AMV reverse transcriptase. The reaction was incubated at 42 °C for 1 h, then the enzyme was inactivated at 70 °C for 10 min. The qRT-PCR was performed in a 25-μL reaction containing 12.5 μL of Applied Biosystems SYBR Premix Ex Taq (2X) (Life Technologies), 1.0 μL of forward primer, 1.0 μL of reverse primer, 1.0 μL of cDNA template, and added nuclease-free water to 25 μL. The reaction program was as follows: preheating at 95 °C for 30 s, 40 cycles of 95 °C for 10 s and 60 °C for 35 s. The fluorescence readings were obtained after each cycle. Finally, the melting curve analysis was performed after the amplification cycles were completed. In order to detect the presence of DNA contamination in the RNA samples, three randomly selected RNA samples were used as templates to perform qRT-PCR as above. The real-time PCR primers were designed using Primer Express 3.0 software (Life Technologies). Glyceraldehyde-3-phosphate dehydrogenase, (GAPDH, NCBI accession no. NM_001111943) was used as an internal reference gene. The qRT-PCR reactions were carried out on a 7500 Real-Time PCR System (Life Technologies). Primers used for qRT-PCR are listed in Table 2. The results were analyzed by the 2^−ΔΔCT^ method^16^.

## Additional Information

**How to cite this article**: Yang, F. *et al.* Analysis of key genes of jasmonic acid mediated signal pathway for defense against insect damages by comparative transcriptome sequencing. *Sci. Rep.* **5**, 16500; doi: 10.1038/srep16500 (2015).

## Supplementary Material

Supplementary Information

## Figures and Tables

**Table 1:** Summary of sequencing data and the statistics of the transcriptome assembly.

Statistical analysis	Four treatments			
	Corn1	CornJA1	CornJAOf2	CornOf2
Raw reads (No.)	53,089,614	62,189,006	61,528,872	75,612,000
Raw bases (bp)	5,362,051,014	6,281,089,606	6,214,416,072	7,636,812,000
≥Q20 (%)	92.39	92.38	92.46	92.39
Clean reads (No.)	48,551,508	56,440,612	55,856,974	68,630,658
Clean bases (bp)	4,649,917,709	5,415,803,095	5,364,910,717	6,587,419,456
≥Q20 (%)	97.91	97.93	97.95	97.95
Mapped sequences (No.)	41,340,146	47,383,413	45,284,950	57,085,043
Mapped bases (bp)	4,175,354,746	4,785,724,713	4,573,779,950	5,765,589,343
Mapped percentage (%)	85.15	83.95	81.07	83.18

Notes: the sequence length is 2 * 101 bp, e.g., each read is 101 bp, paired-end sequencing; mapped percentage = mapped sequences/clean reads.

**Table 2:** Primers for transcriptome sequencing and qRT-PCR.

Genes	Primers for transcriptome sequencing (5′→3′)^a^	Length (bp)	Primers for qRT-PCR (5′→3′)^b^	Length (bp)
*LOX5*	P1:GCCAAGAACACCCGTATCCC	760	F:TCGCGTCTACCGTTACGACTACTA	180
	P2:GGCTTCAGCGTGCCATCGTC		R:TTCAGGTTCAGCAGGAAAAGC	
*BBTI2*	P1:CAAGGACCCGATTATCAGCA	327	F:TGGGACTGCTGCGACTTCG	135
	P2:CCAGGATAGCCGTGGAAGAC		R:GCATCGGTAGCCAGGAGGG	
*BBTI11*	P1:CAGCACTCTGTTGGCGATCC	239	F:GCCGACGACGAAGAAGCA	95
	P2:CGCAGTTTTCGGGGAGGAAG		R:GCCGACGACGAAGAAGCA	
*BBTI12*	P1:GGTGCTGATCCTGTGCCTCCA	213	F:GGCTAAAGAAAATGCCCTGGTT	200
	P2:ACGGCGACGACGCACTTCTTG		R:CGGTGCACCTGAACTTGTTG	
*BBTI13*	P1:GCACGAGGAGGAGGAGGAGGAA	221	F:TTCGCCGTCCTCGTAGCT	150
	P2:GGCGCTTGCAGAAGTTGACGAT		R:TCCCTGCACTCGCATATCG	
*CI-1B*	P1:CGTGGAGGACGCCAAGAAGGT	116	F:AAGAAGGTGATCCTCAAGGACAAG	100
	P2:CGAAGATGCGGACACGGTTAG		R:AAGATGCGGACACGGTTAGG	
*TPS10*	P1:AAGCCATTACCTTCACCA	626	F:ATCTCACCCTTCAAACCCC	168
	P2:TCCTCGGACTAACACCTT		R:TAACCTCTTTCAACTCCTCAC	
*DOX*	P1:GGAAGAACATCCCAACCTA	755	F:GGATTCGTTTTCGTTCATGCAT	473
	P2:CTGCCATAAGACCAACAAG		R:TCCGGTGCGGTACATGAACT	
*A20/AN1*	P1:ACCCATCCTCTGCATCAATA	344	F:CTGGCTGCCTCCTCTATTGACA	197
	P2:ATCCCGTAAGTCCAACCCTC		R:TTCGGCCCTACCTTCCCC	
*GAPDH*	—		P1:CACGGCCACTGGAAGCA	
	—		P2:TCCTCAGGGTTCCTGATGCC	
*P450-72A1*	P1:CAGCACATCGCCTTCTCCACCATTT	652	F:CGCCTTCTCCACCATTTTCAA	178
	P2:TCGTCACCCCGTTCTCGAACCTCTC		R:GAGCCCGAGCAGGTCGTTCCC	
*CYP72A5*	P1: TTTGGGAGCAACTATCAAGAAGGG	870	F: GGTGCTCAGATAAGGCTCAA	186
	P2: GTGCAGTGTTATCACGGTGTACGG		R: ACATCGCAATGCATACAAGG	
*P450-a*	P1: GCATGTCCCCCGTCATCTGC	995	F: TCGCCGTGCTGCTCTCTTTGC	180
	P2: TCCATCAACCGTGTCCCAGT		R: CGATCTCTGCCTGTGCCTTCT	
P450-c	P1: GCTTCAGCGAGGGGAGGAGGATTTT	839	F: AGAGGCATCGTCAGCAAGCG	178
	P2: GTGAGTGTAGGACTGCGAGAGGGCG		R: TCCCGGCGAAGTAGAACAGC	
P450-e	P1: ACAAACCACACACCCCACCTACCCC	541	F: ACGCTGTGGTCGATCGAGTG	149
	P2: GTCCTTCCTCACGTCCTCCACCACC		R: ATGGCCTGAAGGTAGGGGAG	
P450-g	P1: CGACGCAACTGAACCGATCGATGGA	705	F: TACAAGAAGCGGGGAGAGGAA	137
	P2: TGACGGCAGGGAGGAAGGCGAAGAC		R: TCGGAGCATAGGAGGATAATC	
*AN17*	P1: TCCTCTCGTCGCTCGCTCATCACCC	333	F: CCCAATCAATCGCTAACAA	159
	P2: CGCCCTGCCCCGGAACCAGCCTCTC		R: AGTCGAAGGGCAGGAAGTC	
*GPX*	P1: CTCGGCTTCTTCTACACGCTAC	360	F: ACAGCCGTAAACGCCCCTCCC	143
	P2: ACTGGACAATCTCCTCATTGGT		R: TCAACGTCTTTGCCGCTCGCA	

Notes:

^a^P1 stands for forward primers, and P2 stands for reverse primers for transcriptome sequencing.

^b^F stand for forward primers, and R stands for reverse primers for qRT-PCR analysis. GAPDH (Accession no. NM_001111943) is an internal reference gene for qRT-PCR.

**Table 3:** Alternative splicing analysis.

Possible alternative splicing	Corn1	CornJA1	CornOf2	CornJAOf2
3S	3,443	4,155 (20.68%)	4,756 (38.14%)	3,982 (15.65%)
3UTR	1,171	1,240 (5.89%)	1,457 (24.42%)	1,194 (1.96%)
5S	2,217	2,682 (20.97%)	2,945 (32.84%)	2,640 (19.08%)
5UTR	922	1,016 (10.20%)	1,167 (26.57%)	983 (6.62%)
SE	812	523 (−35.59%)	700 (−13.79%)	491 (−39.53%)
RI	990	1,208 (22.02%)	1,459 (47.37%)	1,156 (16.77%)
other	653	873 (33.69%)	977 (49.61%)	850 (30.17%)

Note: 3S: 3′ alternative splice; 5S: 5′ alternative splice; 3UTR: alternative 3′ UTR splice; 5UTR: alternative 5′ UTR splice; SE: skipped exon; RI: retained intron; other: other possible alternative splice. The data in the parentheses are the percent changes of alternatively spliced transcripts compared with control group (Corn1).

**Table 4:** Single nucleotide polymorphisms.

Type	Corn1		CornJA1		CornOf2		CornJAOf2	
	Count	Frequency per kb	Count	Frequency per kb	Count	Frequency per kb	Count	Frequency per kb
Transition								
C/T	66,095	0.82	71,681 (8.45%)	0.89 (8.54%)	80,363 (21.59%)	1 (21.95%)	73,667 (11.46%)	0.91 (10.98%)
A/G	66,328	0.82	71,824 (8.29%)	0.89 (8.54%)	80,626 (21.56%)	1 (21.95%)	73,673 (11.07%)	0.91 (10.98%)
Transversion								
A/T	15,582	0.19	16,296 (4.58%)	0.2 (5.26%)	18,576 (19.21%)	0.23 (21.05%)	16,888 (8.38%)	0.21 (10.53%)
A/C	19,456	0.24	20,239 (4.02%)	0.25 (4.17%)	23,207 (19.28%)	0.28 (16.67%)	20,732 (6.56%)	0.25 (4.17%)
T/G	19,775	0.24	20,375 (3.03%)	0.25 (4.17%)	23,273 (17.69%)	0.28 (16.67%)	20,778 (5.07%)	0.25 (4.17%)
C/G	22,627	0.28	22,219 (−1.80%)	0.27 (−3.57%)	25,757 (13.83%)	0.32 (14.29%)	22,419 (−0.92%)	0.27 (−3.57%)
Total	209,863	2.61	222,634 (6.08%)	2.77 (2.77%)	251,802 (19.98%)	3.13 (19.92%)	228,157 (8.72%)	2.84 (8.81%)

Note: The data in the parentheses are the percent changes compared with control group (Corn1).
