# Supplementary material for: Analysis of key genes of jasmonic acid mediated signal pathway for defense against insect damages by comparative transcriptome sequencing
Source: Sci Rep. 2015 Nov 12;5:16500. doi: 10.1038/srep16500 (PMC4642351; doi:10.1038/srep16500)

Analysis of key genes of jasmonic acid mediated signal pathway for defense against insect damages by comparative transcriptome sequencing

Fengshan Yang[1#], Yuliang Zhang[2#], Qixing Huang[2], Guohua Yin[2,3]*, Kayla K. Pennerman[3], Jiujiang Yu[4], Zhixin Liu[2], Dafei Li[1], Anping Guo[2]*

[1]Key Laboratory of Molecular Biology of Heilongjiang Province, College of Life Sciences, Heilongjiang University, Harbin, Heilongjiang 150080, China

[2]Key Laboratory of Biology and Genetic Resources of Tropical Crops, Ministry of Agriculture, Institute of Tropical Bioscience and Biotechnology, Chinese Academy of Tropical Agricultural Sciences, Haikou, Hainan 571101, China

[3]Department of Plant Biology and Pathology, Rutgers, The State University of New Jersey, New Brunswick, New Jersey 08901, United States

[4]Department of Agriculture, ARS, Beltsville Agricultural Research Center, Beltsville, Maryland 20705, USA

*Corresponding authors: Guohua Yin, guohuayin1997@gmail.com; Anping Guo, gap211@126.com

Supplementary tables

Table S1 GO term of CornJA1 vs Corn1

Table S2 GO term of CornOf2 vs Corn1

Table S3 GO term of CornJAOf2 vs Corn1

Table S4 KEGG pathways of CornJA1 vs Corn1

Table S5 KEGG pathways of CornOf2 vs Corn1

Table S6 KEGG pathways of CornJAOf2 vs Corn1

Table S7 Fifteen screened genes involved in maize defense resistance against Asian corn borer feeding

Table S8 Nine up-regulated gene expression patterns identified by both RNA-Seq and qRT-PCR

Table S1 Go term of CornJA1 Vs Corn1

Gene Ontology term	Cluster frequency		Genome frequency of use	
**Biological process**				
biological process	111/177	62.71%	31234/63459	49.22%
metabolic process	81/177	45.76%	21185/63459	33.38%
response to stimulus	47/177	26.55%	8333/63459	13.13%
organonitrogen compound metabolic process	33/177	18.64%	6142/63459	9.68%
response to abiotic stimulus	29/177	16.38%	3267/63459	5.15%
carboxylic acid metabolic process	29/177	16.38%	4725/63459	7.45%
oxoacid metabolic process	29/177	16.38%	4779/63459	7.53%
organic acid metabolic process	29/177	16.38%	4785/63459	7.54%
cellular amino acid metabolic process	27/177	15.25%	3827/63459	6.03%
response to chemical stimulus	25/177	14.12%	3899/63459	6.14%
oxidation-reduction process	23/177	12.99%	3470/63459	5.47%
response to inorganic substance	21/177	11.86%	2207/63459	3.48%
sulfur compound metabolic process	16/177	9.04%	645/63459	1.02%
response to light stimulus	14/177	7.91%	972/63459	1.53%
response to radiation	14/177	7.91%	1016/63459	1.60%
response to metal ion	14/177	7.91%	1470/63459	2.32%
response to endogenous stimulus	14/177	7.91%	1709/63459	2.69%
response to hormone stimulus	14/177	7.91%	1664/63459	2.62%
cellular amide metabolic process	13/177	7.34%	334/63459	0.53%
response to cadmium ion	13/177	7.34%	1242/63459	1.96%
peptide metabolic process	12/177	6.78%	190/63459	0.30%
glutathione metabolic process	12/177	6.78%	152/63459	0.24%
response to karrikin	12/177	6.78%	307/63459	0.48%
glutathione conjugation reaction	12/177	6.78%	82/63459	0.13%
cellular modified amino acid metabolic process	12/177	6.78%	378/63459	0.60%

response to other organism	12/177	6.78%	1284/63459	2.02%
response to biotic stimulus	12/177	6.78%	1388/63459	2.19%
secondary metabolic process	8/177	4.52%	364/63459	0.57%
response to water stimulus	8/177	4.52%	515/63459	0.81%
response to organic nitrogen	7/177	3.95%	103/63459	0.16%
pigment biosynthetic process	7/177	3.95%	224/63459	0.35%
secondary metabolite biosynthetic process	7/177	3.95%	258/63459	0.41%
pigment metabolic process	7/177	3.95%	290/63459	0.46%
post-embryonic development	7/177	3.95%	514/63459	0.81%
response to peptide hormone stimulus	6/177	3.39%	34/63459	0.05%
response to peptide	6/177	3.39%	34/63459	0.05%
response to growth hormone stimulus	6/177	3.39%	34/63459	0.05%
de-etiolation	6/177	3.39%	41/63459	0.06%
lateral root development	6/177	3.39%	74/63459	0.12%
post-embryonic root development	6/177	3.39%	97/63459	0.15%
phenylpropanoid biosynthetic process	6/177	3.39%	187/63459	0.29%
post-embryonic organ development	6/177	3.39%	199/63459	0.31%
phenylpropanoid metabolic process	6/177	3.39%	270/63459	0.43%
root development	6/177	3.39%	307/63459	0.48%
regulation of hormone levels	6/177	3.39%	313/63459	0.49%
hormone metabolic process	5/177	2.82%	200/63459	0.32%
cellular response to abiotic stimulus	4/177	2.26%	137/63459	0.22%
protein glutathionylation	3/177	1.69%	28/63459	0.04%
brassinosteroid metabolic process	3/177	1.69%	34/63459	0.05%
phytosteroid metabolic process	3/177	1.69%	35/63459	0.06%
flavonoid biosynthetic process	3/177	1.69%	45/63459	0.07%
stilbene metabolic process	3/177	1.69%	57/63459	0.09%
stilbene biosynthetic process	3/177	1.69%	57/63459	0.09%
coumarin biosynthetic process	3/177	1.69%	58/63459	0.09%

coumarin metabolic process	3/177	1.69%	58/63459	0.09%
siroheme metabolic process	2/177	1.13%	9/63459	0.01%
siroheme biosynthetic process	2/177	1.13%	9/63459	0.01%
**Cellular component**				
extracellular region	11/177	6.21%	1241/63459	1.96%
**Molecular function**				
catalytic activity	86/177	48.59%	20322/63459	32.02%
transferase activity	47/177	26.55%	7743/63459	12.20%
oxidoreductase activity	23/177	12.99%	3457/63459	5.45%
transferase activity, transferring glycosyl groups	17/177	9.60%	1337/63459	2.11%
transferase activity, transferring hexosyl groups	15/177	8.47%	932/63459	1.47%
transferase activity, transferring alkyl or aryl (other than methyl) groups	14/177	7.91%	267/63459	0.42%
glutathione transferase activity	12/177	6.78%	82/63459	0.13%
iron ion binding	10/177	5.65%	739/63459	1.16%
oxidoreductase activity, acting on paired donors, with incorporation or reduction of molecular oxygen	9/177	5.08%	783/63459	1.23%
monooxygenase activity	8/177	4.52%	483/63459	0.76%
quercetin 7-O-glucosyltransferase activity	4/177	2.26%	16/63459	0.03%
quercetin 3-O-glucosyltransferase activity	3/177	1.69%	14/63459	0.02%
quercetin 4'-O-glucosyltransferase activity	3/177	1.69%	18/63459	0.03%
precorrin-2 dehydrogenase activity	2/177	1.13%	4/63459	0.01%
chitin binding	2/177	1.13%	15/63459	0.02%
carbohydrate derivative binding	2/177	1.13%	15/63459	0.02%
trans-zeatin O-beta-D-glucosyltransferase activity	2/177	1.13%	15/63459	0.02%

Table S2 Go term of CornOf2 Vs Corn1

Gene Ontology term	Cluster frequency		Genome frequency of use	
**Biological process**				
biological process	134/200	67%	31234/63459	49.22%
metabolic process	98/200	49%	21185/63459	33.38%
response to stimulus	50/200	25%	8333/63459	13.13%
oxidation-reduction process	36/200	18%	3470/63459	5.47%
carboxylic acid metabolic process	31/200	15.50%	4725/63459	7.45%
oxoacid metabolic process	31/200	15.50%	4779/63459	7.53%
organic acid metabolic process	31/200	15.50%	4785/63459	7.54%
response to stress	30/200	15%	4692/63459	7.39%
single-organism biosynthetic process	20/200	10%	1991/63459	3.14%
sulfur compound metabolic process	16/200	8%	645/63459	1.02%
electron transport	16/200	8%	1029/63459	1.62%
response to endogenous stimulus	15/200	7.50%	1709/63459	2.69%
response to hormone stimulus	14/200	7%	1664/63459	2.62%
response to other organism	13/200	6.50%	1284/63459	2.02%
response to biotic stimulus	13/200	6.50%	1388/63459	2.19%
cellular amide metabolic process	11/200	5.50%	334/63459	0.53%
glutathione conjugation reaction	9/200	4.50%	82/63459	0.13%
glutathione metabolic process	9/200	4.50%	152/63459	0.24%
peptide metabolic process	9/200	4.50%	190/63459	0.30%
response to karrikin	9/200	4.50%	307/63459	0.48%
secondary metabolic process	9/200	4.50%	364/63459	0.57%
cellular modified amino acid metabolic process	9/200	4.50%	378/63459	0.60%
response to wounding	8/200	4%	322/63459	0.51%
secondary metabolite biosynthetic process	7/200	3.50%	258/63459	0.41%
hormone metabolic process	6/200	3%	200/63459	0.32%
pigment biosynthetic process	6/200	3%	224/63459	0.35%

pigment metabolic process	6/200	3%	290/63459	0.46%
regulation of hormone levels	6/200	3%	313/63459	0.49%
benzene-containing compound metabolic process	5/200	2.50%	100/63459	0.16%
response to organic nitrogen	5/200	2.50%	103/63459	0.16%
phenylpropanoid biosynthetic process	5/200	2.50%	187/63459	0.29%
steroid metabolic process	5/200	2.50%	215/63459	0.34%
cellular response to extracellular stimulus	5/200	2.50%	219/63459	0.35%
cellular response to external stimulus	5/200	2.50%	219/63459	0.35%
S-glycoside metabolic process	4/200	2%	48/63459	0.08%
glycosinolate metabolic process	4/200	2%	48/63459	0.08%
glucosinolate metabolic process	4/200	2%	48/63459	0.08%
benzoate metabolic process	4/200	2%	53/63459	0.08%
cellular response to abiotic stimulus	4/200	2%	137/63459	0.22%
toluene metabolic process	3/200	1.50%	23/63459	0.04%
toluene-containing compound metabolic process	3/200	1.50%	23/63459	0.04%
toluene-containing compound catabolic process	3/200	1.50%	23/63459	0.04%
xylene catabolic process	3/200	1.50%	23/63459	0.04%
xylene metabolic process	3/200	1.50%	23/63459	0.04%
toluene catabolic process	3/200	1.50%	23/63459	0.04%
response to peptide hormone stimulus	3/200	1.50%	34/63459	0.05%
brassinosteroid metabolic process	3/200	1.50%	34/63459	0.05%
response to peptide	3/200	1.50%	34/63459	0.05%
response to growth hormone stimulus	3/200	1.50%	34/63459	0.05%
phytosteroid metabolic process	3/200	1.50%	35/63459	0.06%
jasmonic acid biosynthetic process	3/200	1.50%	41/63459	0.06%
de-etiolation	3/200	1.50%	41/63459	0.06%
flavonoid biosynthetic process	3/200	1.50%	45/63459	0.07%
xenobiotic catabolic process	3/200	1.50%	47/63459	0.07%
jasmonic acid metabolic process	3/200	1.50%	51/63459	0.08%

oxylipin metabolic process	3/200	1.50%	58/63459	0.09%
siroheme metabolic process	2/200	1%	9/63459	0.01%
siroheme biosynthetic process	2/200	1%	9/63459	0.01%
**Molecular function**				
molecular_function	135/200	67.50%	34124/63459	53.77%
catalytic activity	100/200	50%	20322/63459	32.02%
transferase activity	41/200	20.50%	7743/63459	12.20%
oxidoreductase activity	36/200	18%	3457/63459	5.45%
metal ion binding	27/200	13.50%	4268/63459	6.73%
transition metal ion binding	22/200	11%	1617/63459	2.55%
iron ion binding	18/200	9%	739/63459	1.16%
oxidoreductase activity, acting on paired donors, with incorporation or reduction of molecular oxygen	16/200	8%	783/63459	1.23%
electron carrier activity	16/200	8%	833/63459	1.31%
heme binding	14/200	7%	725/63459	1.14%
tetrapyrrole binding	14/200	7%	757/63459	1.19%
transferase activity, transferring glycosyl groups	13/200	6.50%	1337/63459	2.11%
monooxygenase activity	12/200	6%	483/63459	0.76%
transferase activity, transferring hexosyl groups	12/200	6%	932/63459	1.47%
transferase activity, transferring alkyl or aryl (other than methyl) groups	10/200	5%	267/63459	0.42%
glutathione transferase activity	9/200	4.50%	82/63459	0.13%
dioxygenase activity	8/200	4%	361/63459	0.57%
oxidoreductase activity, acting on single donors with incorporation of molecular oxygen, incorporation of two atoms of oxygen	5/200	2.50%	137/63459	0.22%
oxidoreductase activity, acting on single donors with incorporation of molecular oxygen	5/200	2.50%	155/63459	0.24%
quercetin 7-O-glucosyltransferase activity	4/200	2%	16/63459	0.03%

oxidoreductase activity, acting on the CH-CH group of donors, NAD or NADP as acceptor	4/200	2%	73/63459	0.12%
quercetin 3-O-glucosyltransferase activity	3/200	1.50%	14/63459	0.02%
quercetin 4'-O-glucosyltransferase activity	3/200	1.50%	18/63459	0.03%
precorrin-2 dehydrogenase activity	2/200	1%	4/63459	0.01%
12-oxophytodienoate reductase activity	2/200	1%	7/63459	0.01%
FAD binding	2/200	1%	8/63459	0.01%
oxidoreductase activity, acting on the aldehyde or oxo group of donors, oxygen as acceptor	2/200	1%	13/63459	0.02%
aldehyde oxidase activity	2/200	1%	13/63459	0.02%
benzaldehyde dehydrogenase (NAD+) activity	2/200	1%	13/63459	0.02%
abscisic aldehyde oxidase activity	2/200	1%	13/63459	0.02%
aryl-aldehyde oxidase activity	2/200	1%	13/63459	0.02%
indole-3-acetaldehyde oxidase activity	2/200	1%	13/63459	0.02%
molybdopterin cofactor binding	2/200	1%	14/63459	0.02%

38

Table S3 Go term of CornJAOf2 Vs Corn1

Gene Ontology term	Cluster frequency		Genome frequency of use	
**Biological process**				
biological process	150/240	62.50%	31234/63459	49.22%
metabolic process	109/240	45.42%	21185/63459	33.38%
response to stimulus	58/240	24.17%	8333/63459	13.13%
biosynthetic process	40/240	16.67%	6128/63459	9.66%
cellular biosynthetic process	38/240	15.83%	5531/63459	8.72%
oxidation-reduction process	37/240	15.42%	3470/63459	5.47%
oxoacid metabolic process	36/240	15%	4779/63459	7.53%
organic acid metabolic process	36/240	15%	4785/63459	7.54%
carboxylic acid metabolic process	35/240	14.58%	4725/63459	7.45%
response to abiotic stimulus	33/240	13.75%	3267/63459	5.15%
response to stress	33/240	13.75%	4692/63459	7.39%
response to chemical stimulus	31/240	12.92%	3899/63459	6.14%
single-organism biosynthetic process	25/240	10.42%	1991/63459	3.14%
response to inorganic substance	21/240	8.75%	2207/63459	3.48%
small molecule biosynthetic process	18/240	7.50%	1827/63459	2.88%
response to biotic stimulus	17/240	7.08%	1388/63459	2.19%
response to hormone stimulus	17/240	7.08%	1664/63459	2.62%
response to endogenous stimulus	17/240	7.08%	1709/63459	2.69%
sulfur compound metabolic process	15/240	6.25%	645/63459	1.02%
carboxylic acid biosynthetic process	15/240	6.25%	1396/63459	2.20%
organic acid biosynthetic process	15/240	6.25%	1396/63459	2.20%
response to light stimulus	14/240	5.83%	972/63459	1.53%
response to radiation	14/240	5.83%	1016/63459	1.60%
monocarboxylic acid metabolic process	14/240	5.83%	1127/63459	1.78%
response to other organism	14/240	5.83%	1284/63459	2.02%

cellular amide metabolic process	11/240	4.58%	334/63459	0.53%
response to water stimulus	11/240	4.58%	515/63459	0.81%
cellular modified amino acid metabolic process	10/240	4.17%	378/63459	0.60%
secondary metabolic process	10/240	4.17%	364/63459	0.57%
monocarboxylic acid biosynthetic process	10/240	4.17%	625/63459	0.98%
response to cold	10/240	4.17%	713/63459	1.12%
glutathione conjugation reaction	9/240	3.75%	82/63459	0.13%
glutathione metabolic process	9/240	3.75%	152/63459	0.24%
peptide metabolic process	9/240	3.75%	190/63459	0.30%
pigment biosynthetic process	9/240	3.75%	224/63459	0.35%
pigment metabolic process	9/240	3.75%	290/63459	0.46%
response to karrikin	9/240	3.75%	307/63459	0.48%
response to water deprivation	9/240	3.75%	479/63459	0.75%
secondary metabolite biosynthetic process	8/240	3.33%	258/63459	0.41%
phenylpropanoid metabolic process	8/240	3.33%	270/63459	0.43%
post-embryonic development	8/240	3.33%	514/63459	0.81%
phenylpropanoid biosynthetic process	7/240	2.92%	187/63459	0.29%
response to fungus	7/240	2.92%	359/63459	0.57%
response to organic nitrogen	5/240	2.08%	103/63459	0.16%
response to peptide hormone stimulus	4/240	1.67%	34/63459	0.05%
response to peptide	4/240	1.67%	34/63459	0.05%
response to growth hormone stimulus	4/240	1.67%	34/63459	0.05%
de-etiolation	4/240	1.67%	41/63459	0.06%
flavonoid biosynthetic process	4/240	1.67%	45/63459	0.07%
lateral root development	4/240	1.67%	74/63459	0.12%
flavonoid metabolic process	4/240	1.67%	86/63459	0.14%
post-embryonic root development	4/240	1.67%	97/63459	0.15%
jasmonic acid biosynthetic process	3/240	1.25%	41/63459	0.06%
siroheme metabolic process	2/240	0.83%	9/63459	0.01%

siroheme biosynthetic process	2/240	0.83%	9/63459	0.01%
L-asparagine biosynthetic process	2/240	0.83%	11/63459	0.02%
L-asparagine metabolic process	2/240	0.83%	11/63459	0.02%
cuticle hydrocarbon biosynthetic process	2/240	0.83%	11/63459	0.02%
**Cellular component**				
extracellular region	17/240	7.08%	1241/63459	1.96%
**Molecular function**				
catalytic activity	115/240	47.92%	20322/63459	32.02%
transferase activity	51/240	21.25%	7743/63459	12.20%
oxidoreductase activity	37/240	15.42%	3457/63459	5.45%
transferase activity, transferring glycosyl groups	20/240	8.33%	1337/63459	2.11%
transition metal ion binding	19/240	7.92%	1617/63459	2.55%
transferase activity, transferring hexosyl groups	18/240	7.50%	932/63459	1.47%
iron ion binding	17/240	7.08%	739/63459	1.16%
oxidoreductase activity, acting on paired donors, with incorporation or reduction of molecular oxygen	12/240	5%	783/63459	1.23%
transferase activity, transferring alkyl or aryl (other than methyl) groups	10/240	4.17%	267/63459	0.42%
glutathione transferase activity	9/240	3.75%	82/63459	0.13%
dioxygenase activity	9/240	3.75%	361/63459	0.57%
monooxygenase activity	8/240	3.33%	483/63459	0.76%
quercetin 7-O-glucosyltransferase activity	4/240	1.67%	16/63459	0.03%
oxidoreductase activity, acting on the CH-CH group of donors, NAD or NADP as acceptor	4/240	1.67%	73/63459	0.12%
serine-type endopeptidase inhibitor activity	4/240	1.67%	75/63459	0.12%
endopeptidase regulator activity	4/240	1.67%	97/63459	0.15%
peptidase regulator activity	4/240	1.67%	97/63459	0.15%
endopeptidase inhibitor activity	4/240	1.67%	97/63459	0.15%
peptidase inhibitor activity	4/240	1.67%	97/63459	0.15%

quercetin 3-O-glucosyltransferase activity	3/240	1.25%	14/63459	0.02%
quercetin 4'-O-glucosyltransferase activity	3/240	1.25%	18/63459	0.03%
inositol 3-alpha-galactosyltransferase activity	2/240	0.83%	3/63459	0.00%
precorrin-2 dehydrogenase activity	2/240	0.83%	4/63459	0.01%
12-oxophytodienoate reductase activity	2/240	0.83%	7/63459	0.01%
glutamyl-tRNA reductase activity	2/240	0.83%	7/63459	0.01%

Table S4 KEGG pathways of CornJA1 Vs Corn1

KEGG Pathway Term	ID	Sample No.	Background No.	P Value	Corrected	Genes	Hyperlink
Metabolic pathways	zma01100	7	3804	0.488524186	0.489505774	PAC:20850353(GRMZM2G069203) |PAC:20880541(GRMZM2G107402) |PAC:20882175(GRMZM2G069542) |PAC:20823765(GRMZM2G306345) |PAC:20830586(GRMZM2G339699) |PAC:20867951(GRMZM2G131205) |PAC:20847248(GRMZM2G365160)	http://www.genome.jp/kegg-bin/show_pathway?zma01100
Biosynthesis of secondary metabolites	zma01110	5	1751	0.1777703	0.330149105	PAC:20830586(GRMZM2G339699) |PAC:20880541(GRMZM2G107402) |PAC:20867951(GRMZM2G131205) |PAC:20824454(GRMZM2G163809) |PAC:20847248(GRMZM2G365160)	http://www.genome.jp/kegg-bin/show_pathway?zma01110
Glutathione metabolism	zma00480	4	166	0.000181184	0.003261307	PAC:20871316(GRMZM2G044383) |PAC:20866919(GRMZM2G480439) |PAC:20868308(GRMZM2G042639) |PAC:20863364(GRMZM2G043291)	http://www.genome.jp/kegg-bin/show_pathway?zma00480
Pyruvate metabolism	zma00620	2	190	0.042070599	0.289348432	PAC:20882175(GRMZM2G069542) |PAC:20823765(GRMZM2G306345)	http://www.genome.jp/kegg-bin/show_pathway?zma00620
Carbon fixation in photosynthetic organisms	zma00710	2	205	0.048224739	0.289348432	PAC:20882175(GRMZM2G069542) |PAC:20823765(GRMZM2G306345)	http://www.genome.jp/kegg-bin/show_pathway?zma00710
Tryptophan metabolism	zma00380	1	71	0.115147283	0.330149105	PAC:20865680(GRMZM2G141535)	http://www.genome.jp/kegg-bin/show_pathway?zma00380
Sulfur metabolism	zma00920	1	85	0.136282819	0.330149105	PAC:20850353(GRMZM2G069203)	http://www.genome.jp/kegg-bin/show_pathway?zma00920
Terpenoid backbone biosynthesis	zma00900	1	116	0.181355382	0.330149105	PAC:20824454(GRMZM2G163809)	http://www.genome.jp/kegg-bin/show_pathway?zma00900
Porphyrin and chlorophyll metabolism	zma00860	1	116	0.181355382	0.330149105	PAC:20880541(GRMZM2G107402)	http://www.genome.jp/kegg-bin/show_pathway?zma00860
Phenylalanine, tyrosine and tryptophan biosynthesis	zma00400	1	128	0.198183238	0.330149105	PAC:20847248(GRMZM2G365160)	http://www.genome.jp/kegg-bin/show_pathway?zma00400
Alanine, aspartate and glutamate metabolism	zma00250	1	130	0.200955073	0.330149105	PAC:20830586(GRMZM2G339699)	http://www.genome.jp/kegg-bin/show_pathway?zma00250
Phenylpropanoid biosynthesis	zma00940	1	132	0.203717614	0.330149105	PAC:20867951(GRMZM2G131205)	http://www.genome.jp/kegg-bin/show_pathway?zma00940
Galactose metabolism	zma00052	1	144	0.220099404	0.330149105	PAC:20825959(GRMZM2G150906)	http://www.genome.jp/kegg-bin/show_pathway?zma00052
Ribosome biogenesis in eukaryotes	zma03008	1	198	0.289860981	0.401345973	PAC:20862149(GRMZM2G169931)	http://www.genome.jp/kegg-bin/show_pathway?zma03008

Cysteine and methionine metabolism	zma00270	1	218	0.314130556	0.403882143	PAC:20850353(GRMZM2G069203)	http://www.genome.jp/kegg-bin/show_pathway?zma00270
Purine metabolism	zma00230	1	349	0.454332067	0.489505774	PAC:20830586(GRMZM2G339699)	http://www.genome.jp/kegg-bin/show_pathway?zma00230
RNA transport	zma03013	1	357	0.461927049	0.489505774	PAC:20862149(GRMZM2G169931)	http://www.genome.jp/kegg-bin/show_pathway?zma03013
Plant hormone signal transduction	zma04075	1	387	0.489505774	0.489505774	PAC:20826302(GRMZM2G177220)	http://www.genome.jp/kegg-bin/show_pathway?zma04075

Table S5 KEGG pathways of CornOf2 Vs Corn1

KEGG Pathway Term	ID	Sample No.	Background No.	P Value	Corrected	Genes	Hyperlink
Metabolic pathways	zma01100	10	3804	0.4864599	0.547267388	PAC:20862762(GRMZM2G053669) |PAC:20851783(GRMZM2G327427) |PAC:20830085(GRMZM2G026182) |PAC:20830586(GRMZM2G339699) |PAC:20847955(GRMZM2G158147) |PAC:20867951(GRMZM2G131205) |PAC:20830527(GRMZM2G156861) |PAC:20880949(GRMZM2G139300) |PAC:20836281(GRMZM2G493395) |PAC:20869899(GRMZM2G002178)	http://www.genome.jp/kegg-bin/show_pathway?zma01100
Biosynthesis of secondary metabolites	zma01110	6	1751	0.270713271	0.423206641	PAC:20862762(GRMZM2G053669) |PAC:20830085(GRMZM2G026182) |PAC:20830586(GRMZM2G339699) |PAC:20867951(GRMZM2G131205) |PAC:20824454(GRMZM2G163809) |PAC:20836281(GRMZM2G493395)	http://www.genome.jp/kegg-bin/show_pathway?zma01110
Linoleic acid metabolism	zma00591	2	33	0.003074298	0.083006048	PAC:20878337(GRMZM2G102760) |PAC:20830527(GRMZM2G156861)	http://www.genome.jp/kegg-bin/show_pathway?zma00591
alpha-Linolenic acid metabolism	zma00592	2	83	0.018305532	0.247124684	PAC:20830527(GRMZM2G156861) |PAC:20869899(GRMZM2G002178)	http://www.genome.jp/kegg-bin/show_pathway?zma00592
Terpenoid backbone biosynthesis	zma00900	2	116	0.034064949	0.263756469	PAC:20824454(GRMZM2G163809) |PAC:20836281(GRMZM2G493395)	http://www.genome.jp/kegg-bin/show_pathway?zma00900
Alanine, aspartate and glutamate metabolism	zma00250	2	130	0.041895013	0.263756469	PAC:20830586(GRMZM2G339699) |PAC:20862762(GRMZM2G053669)	http://www.genome.jp/kegg-bin/show_pathway?zma00250
Glutathione metabolism	zma00480	2	166	0.064690905	0.291109074	PAC:20871316(GRMZM2G044383) |PAC:20863364(GRMZM2G043291)	http://www.genome.jp/kegg-bin/show_pathway?zma00480
Purine metabolism	zma00230	2	349	0.216897754	0.40996329	PAC:20830586(GRMZM2G339699) |PAC:20847955(GRMZM2G158147)	http://www.genome.jp/kegg-bin/show_pathway?zma00230
RNA transport	zma03013	2	357	0.224264842	0.40996329	PAC:20833764(GRMZM2G301904) |PAC:20862149(GRMZM2G169931)	http://www.genome.jp/kegg-bin/show_pathway?zma03013
Benzoxazinoid biosynthesis	zma00402	1	20	0.048843791	0.263756469	PAC:20856241(GRMZM2G567452)	http://www.genome.jp/kegg-bin/show_pathway?zma00402
Selenocompound metabolism	zma00450	1	53	0.124375878	0.40996329	PAC:20847955(GRMZM2G158147)	http://www.genome.jp/kegg-bin/show_pathway?zma00450
SNARE interactions in vesicular transport	zma04130	1	60	0.139625845	0.40996329	PAC:20819962(GRMZM2G417004)	http://www.genome.jp/kegg-bin/show_pathway?zma04130
Tryptophan metabolism	zma00380	1	71	0.163066131	0.40996329	PAC:20865680(GRMZM2G141535)	http://www.genome.jp/kegg-bin/show_pathway?zma00380
Pantothenate and CoA biosynthesis	zma00770	1	78	0.177655876	0.40996329	PAC:20851783(GRMZM2G327427)	http://www.genome.jp/kegg-bin/show_pathway?zma00770
beta-Alanine metabolism	zma00410	1	78	0.177655876	0.40996329	PAC:20851783(GRMZM2G327427)	http://www.genome.jp/kegg-bin/show_pathway?zma00410
Sulfur metabolism	zma00920	1	85	0.191996495	0.40996329	PAC:20847955(GRMZM2G158147)	http://www.genome.jp/kegg-bin/show_pathway?zma00920

Nitrogen metabolism	zma00910	1	103	0.227757383	0.40996329	PAC:20862762(GRMZM2G053669)	http://www.genome.jp/kegg-bin/show_pathway?zma00910
Inositol phosphate metabolism	zma00562	1	116	0.252616144	0.423206641	PAC:20830085(GRMZM2G026182)	http://www.genome.jp/kegg-bin/show_pathway?zma00562
Phenylpropanoid biosynthesis	zma00940	1	132	0.282137761	0.423206641	PAC:20867951(GRMZM2G131205)	http://www.genome.jp/kegg-bin/show_pathway?zma00940
Galactose metabolism	zma00052	1	144	0.303526645	0.431327338	PAC:20880949(GRMZM2G139300)	http://www.genome.jp/kegg-bin/show_pathway?zma00052
Fructose and mannose metabolism	zma00051	1	178	0.360796547	0.487075339	PAC:20830085(GRMZM2G026182)	http://www.genome.jp/kegg-bin/show_pathway?zma00051
Ribosome biogenesis in eukaryotes	zma03008	1	198	0.392302943	0.494550174	PAC:20862149(GRMZM2G169931)	http://www.genome.jp/kegg-bin/show_pathway?zma03008
Carbon fixation in photosynthetic organisms	zma00710	1	205	0.402966808	0.494550174	PAC:20830085(GRMZM2G026182)	http://www.genome.jp/kegg-bin/show_pathway?zma00710
Starch and sucrose metabolism	zma00500	1	261	0.481931824	0.547267388	PAC:20880949(GRMZM2G139300)	http://www.genome.jp/kegg-bin/show_pathway?zma00500
Pyrimidine metabolism	zma00240	1	281	0.507576714	0.548182851	PAC:20851783(GRMZM2G327427)	http://www.genome.jp/kegg-bin/show_pathway?zma00240
Glycolysis / Gluconeogenesis	zma00010	1	322	0.556323463	0.577720519	PAC:20830085(GRMZM2G026182)	http://www.genome.jp/kegg-bin/show_pathway?zma00010
Plant hormone signal transduction	zma04075	1	387	0.624083413	0.624083413	PAC:20826302(GRMZM2G177220)	http://www.genome.jp/kegg-bin/show_pathway?zma04075

Table S6 KEGG pathways of CornJAOf2 Vs Corn1

KEGG Pathway Term	ID	Sample No.	Background No.	P Value	Corrected	Genes	Hyperlink
Metabolic pathways	zma01100	12	3804	0.406137597	0.4984416	PAC:20832956(GRMZM2G025182) |PAC:20862762(GRMZM2G053669) |PAC:20850353(GRMZM2G069203) |PAC:20880541(GRMZM2G107402) |PAC:20847955(GRMZM2G158147) |PAC:20830586(GRMZM2G339699) |PAC:20864552(AC213521.3_FG005) |PAC:20867951(GRMZM2G131205) |PAC:20830527(GRMZM2G156861) |PAC:20865957(GRMZM2G155242) |PAC:20837807(GRMZM2G078472) |PAC:20877852(GRMZM2G029048)	http://www.genome.jp/kegg-bin/show_pathway?zma01100
Biosynthesis of secondary metabolites	zma01110	9	1751	0.059466334	0.22937015	PAC:20862762(GRMZM2G053669) |PAC:20880541(GRMZM2G107402) |PAC:20830586(GRMZM2G339699) |PAC:20864552(AC213521.3_FG005) |PAC:20867951(GRMZM2G131205) |PAC:20824454(GRMZM2G163809) |PAC:20865957(GRMZM2G155242) |PAC:20837807(GRMZM2G078472) |PAC:20877852(GRMZM2G029048)	http://www.genome.jp/kegg-bin/show_pathway?zma01110
Nitrogen metabolism	zma00910	3	103	0.003211111	0.08306132	PAC:20862762(GRMZM2G053669) |PAC:20837807(GRMZM2G078472) |PAC:20877852(GRMZM2G029048)	http://www.genome.jp/kegg-bin/show_pathway?zma00910
Alanine, aspartate and glutamate metabolism	zma00250	3	130	0.00615269	0.08306132	PAC:20830586(GRMZM2G339699) |PAC:20862762(GRMZM2G053669) |PAC:20837807(GRMZM2G078472)	http://www.genome.jp/kegg-bin/show_pathway?zma00250
Sulfur metabolism	zma00920	2	85	0.024706137	0.22235523	PAC:20847955(GRMZM2G158147) |PAC:20850353(GRMZM2G069203)	http://www.genome.jp/kegg-bin/show_pathway?zma00920
Porphyrin and chlorophyll metabolism	zma00860	2	116	0.043646214	0.22937015	PAC:20880541(GRMZM2G107402) |PAC:20864552(AC213521.3_FG005)	http://www.genome.jp/kegg-bin/show_pathway?zma00860
Phenylpropanoid biosynthesis	zma00940	2	132	0.054972748	0.22937015	PAC:20867951(GRMZM2G131205) |PAC:20877852(GRMZM2G029048)	http://www.genome.jp/kegg-bin/show_pathway?zma00940
Purine metabolism	zma00230	2	349	0.264046628	0.42559782	PAC:20830586(GRMZM2G339699) |PAC:20847955(GRMZM2G158147)	http://www.genome.jp/kegg-bin/show_pathway?zma00230
Plant hormone signal transduction	zma04075	2	387	0.304472406	0.43267131	PAC:20873155(GRMZM2G129954) |PAC:20826302(GRMZM2G177220)	http://www.genome.jp/kegg-bin/show_pathway?zma04075

Benzoxazinoid biosynthesis	zma00402	1	20	0.055774582	0.22937015	PAC:20856241(GRMZM2G567452)	http://www.genome.jp/kegg-bin/show_pathway?zma00402
Linoleic acid metabolism	zma00591	1	33	0.090378061	0.30502596	PAC:20830527(GRMZM2G156861)	http://www.genome.jp/kegg-bin/show_pathway?zma00591
Fatty acid elongation	zma00062	1	38	0.103352901	0.3100587	PAC:20869331(GRMZM2G031790)	http://www.genome.jp/kegg-bin/show_pathway?zma00062
Selenocompound metabolism	zma00450	1	53	0.141196853	0.3812315	PAC:20847955(GRMZM2G158147)	http://www.genome.jp/kegg-bin/show_pathway?zma00450
Tryptophan metabolism	zma00380	1	71	0.184543643	0.42559782	PAC:20865680(GRMZM2G141535)	http://www.genome.jp/kegg-bin/show_pathway?zma00380
Carotenoid biosynthesis	zma00906	1	81	0.207689326	0.42559782	PAC:20874809(GRMZM2G152135)	http://www.genome.jp/kegg-bin/show_pathway?zma00906
alpha-Linolenic acid metabolism	zma00592	1	83	0.212240449	0.42559782	PAC:20830527(GRMZM2G156861)	http://www.genome.jp/kegg-bin/show_pathway?zma00592
Phenylalanine metabolism	zma00360	1	103	0.256363045	0.42559782	PAC:20877852(GRMZM2G029048)	http://www.genome.jp/kegg-bin/show_pathway?zma00360
Pentose and glucuronate interconversions	zma00040	1	105	0.260639493	0.42559782	PAC:20832956(GRMZM2G025182)	http://www.genome.jp/kegg-bin/show_pathway?zma00040
Terpenoid backbone biosynthesis	zma00900	1	116	0.283731882	0.42559782	PAC:20824454(GRMZM2G163809)	http://www.genome.jp/kegg-bin/show_pathway?zma00900
Inositol phosphate metabolism	zma00562	1	116	0.283731882	0.42559782	PAC:20865957(GRMZM2G155242)	http://www.genome.jp/kegg-bin/show_pathway?zma00562
Galactose metabolism	zma00052	1	144	0.339364754	0.45814242	PAC:20825959(GRMZM2G150906)	http://www.genome.jp/kegg-bin/show_pathway?zma00052
Glutathione metabolism	zma00480	1	166	0.380079491	0.48867363	PAC:20863364(GRMZM2G043291)	http://www.genome.jp/kegg-bin/show_pathway?zma00480
Ribosome biogenesis in eukaryotes	zma03008	1	198	0.434938141	0.51057956	PAC:20862149(GRMZM2G169931)	http://www.genome.jp/kegg-bin/show_pathway?zma03008
Cysteine and methionine metabolism	zma00270	1	218	0.466773753	0.52512047	PAC:20850353(GRMZM2G069203)	http://www.genome.jp/kegg-bin/show_pathway?zma00270
Starch and sucrose metabolism	zma00500	1	261	0.529375196	0.57172521	PAC:20832956(GRMZM2G025182)	http://www.genome.jp/kegg-bin/show_pathway?zma00500
RNA transport	zma03013	1	357	0.644252397	0.66543998	PAC:20862149(GRMZM2G169931)	http://www.genome.jp/kegg-bin/show_pathway?zma03013
Oxidative phosphorylation	zma00190	1	378	0.665439976	0.66543998	PAC:20849785(GRMZM2G026470)	http://www.genome.jp/kegg-bin/show_pathway?zma00190

Table S7: Fifteen screened up-regulated genes involved in maize defense resistance against Asian corn borer feeding

Category	SEQ ID	Gene name	RNA-Seq (log_2FC)			Function
			CornOf2/Corn1	CornJA1/Corn1	CornJAOf2/Corn1	
JA	GRMZM2G179092	*TPS10*	10.46	-0.08	7.20	terpene synthesis
JA	GRMZM2G156861	*LOX1*	6.866	1.960	4.060	linoleic and α-linolenic metabolism
JA	GRMZM2G156632	*BBTI12*	5.01	0.680	5.620	serine protease inhibitor
SA/JA	GRMZM2G053669	*ASN1*	4.18	2.04	4.77	conversion of Gln and Asp to Asn and Glu
JA	GRMZM2G312997	*DOX*	3.65	2.96	3.72	oxygenation of fatty acids
JA	AC208221.3_FG002	*BBTI2*	3.55	2.61	2.66	serine protease inhibitor
JA	GRMZM2G493395	*DXS*	3.48	-3.50	0.77	1-deoxyxylulose-5-phosphate production
JA	GRMZM2G096680	*CI-1B*	3.29	1.56	4.39	serine protease inhibitor
JA	GRMZM2G102760	*LOX5*	2.98	0.059	1.41	linoleic and α-linolenic metabolism
SA/JA	GRMZM2G301904	*eIF3*	2.87	1.27	1.02	transcription initiation
JA	GRMZM2G002178	*AOS*	2.83	0.28	1.12	octadecanoid synthesis
JA	GRMZM2G011523	*BBTI11*	2.83	-1.81	0.48	serine protease inhibitor
JA	GRMZM2G026182	*TIM*	2.71	1.01	1.01	1-deoxyxylulose-5-phosphate production
JA	GRMZM2G007928	*BBTI13*	2.57	2.01	2.88	serine protease inhibitor
JA	GRMZM5G836222	*A20/AN1*	2.06	1.33	1.84	abiotic stress tolerance

Note: JA: jasmonic acid; SA: salicylic acid; TPS10: terpene synthase 10; LOX1/5: lipoxygenase 1/5; BBTI2/11/12/13: Bowman-Birk type inhibitor 2/11/12/13; ASN1: asparagine synthetase 1; DOX: α-dioxygenase; DXS:1-deoxyxylulose-5-phosphate synthase; CI-1B: subtilisin-chymotrypsin inhibitor CI-1B; eIF3:eukaryotic translation initiation; AOS: allene oxide synthase; TIM: triosephosphate isomerase; A20/AN1: zinc finger A20 and AN1 domain-containing stress-associated protein.

Table S8: Nine up-regulated gene expression patterns identified by both RNA-Seq and qRT-PCR

Gene ID	Genes	Descriptions	Corn1	CornOf2	CorJA1	CornJAOf2	CornOf2 fold changes		CornJA1 fold changes		CornJAOf2 fold changes	
							RNA-Seq	qRT-PCR	RNA-Seq	qRT-PCR	RNA-Seq	qRT-PCR
GRMZM2G102760	*LOX5*	lipoxygenase 5	9.12094	71.9712	9.45743	24.2126	7.9	1.8	1.0	0.9	2.7	2.5
AC208221.3_FG002	*BBTI2*	Bowman-Birk type bran trypsin inhibitor precursor	29.7042	348.324	181.395	187.164	11.7	7.3	6.1	3.0	6.3	3.1
GRMZM2G011523	*BBTI11*		59.7273	424.36	17.0728	83.1047	7.1	9.9	0.3	1.5	1.4	5.1
GRMZM2G156632	*BBTI12*		4.07149	131.088	6.53184	200.857	32.2	34.2	1.6	7.2	49.3	10.5
GRMZM2G007928	*BBTI13*		61.8682	366.857	248.906	456.12	5.9	5.1	4.00	4.7	7.4	15.9
GRMZM2G096680	*CI-1B*	subtilisin-chymotrypsin inhibitor CI-1B	0	9.79193	0.294325	20.9477	-	6.8	-	2.8	-	17.2
GRMZM2G179092	*TPS10*	terpene synthase10	0.10597	149.202	0	15.5579	1408.0	42.6	-	10.3	146.8	31.9
GRMZM2G312997	*DOX*	fatty acid alpha-dioxygenase	2.12431	26.7002	16.5777	27.9404	12.6	18.5	7.8	6.4	13.2	36.8
GRMZM5G836222	*A20/AN1*	zinc finger A20 and AN1 domain-containing stress-associated protein	335.155	1401.45	843.635	1200.43	4.2	5.8	2.5	2.2	3.6	5.2

Supplementary figure

Figure S1: Distribution and classification of genes according to Gene Ontology analyses of DEGs in CornJA1, CornOf2, and CornJAOf2 treatments.

Note: Corn1 was the control group. Analyzed DEGs had at least 10 FPKM in at least one treatment and were at least 2 fold changes.

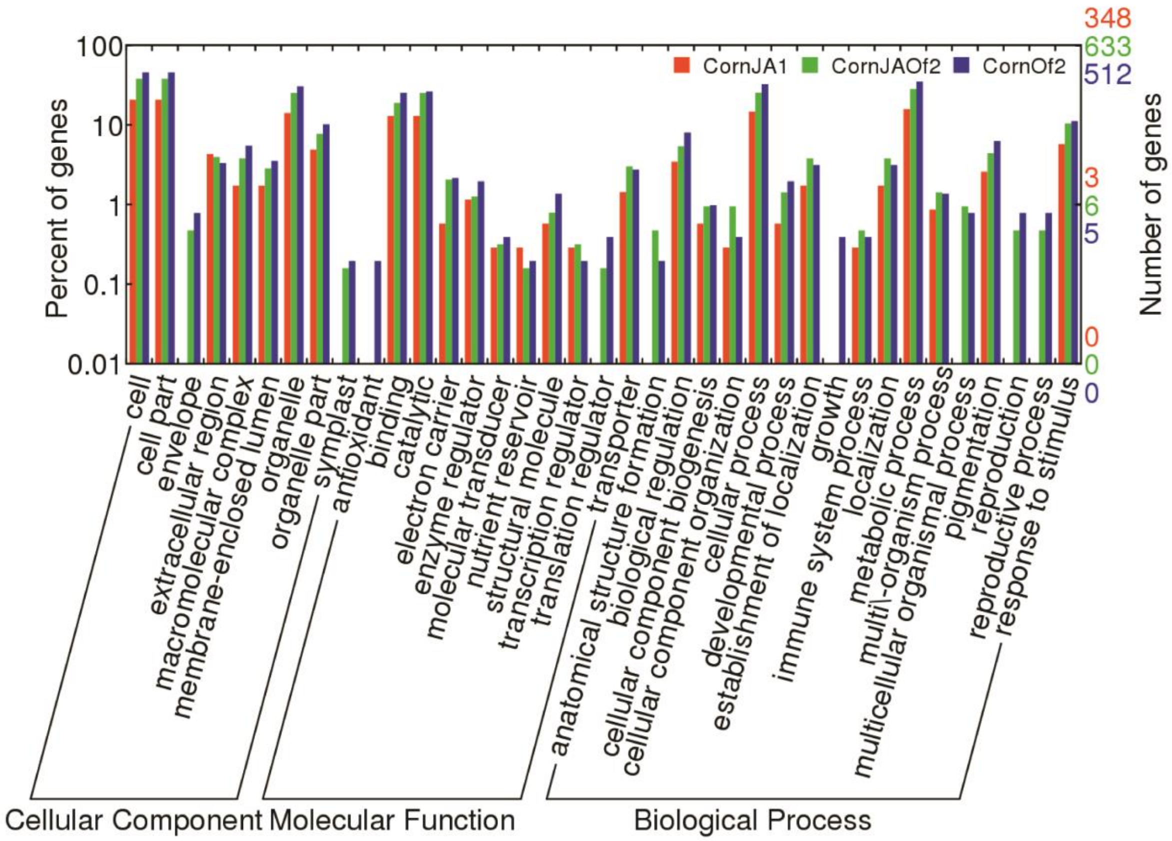

Supplement: Supplementary Information [file srep16500-s1.pdf]
